# Bis(adamantan-1-aminium) carbonate

**DOI:** 10.1107/S1600536812011828

**Published:** 2012-03-24

**Authors:** Monika Nowakowska, Caryn Gamble, Demetrius C. Levendis

**Affiliations:** a Molecular Sciences Institute, School of Chemistry, University of the Witwatersrand, Johannesburg, PO Wits 2050, South Africa

## Abstract

In the title compound, 2C_10_H_18_N^+^·CO_3_^2−^, the adamantan-1-aminium cation forms three N—H⋯O hydrogen bonds to three carbonate ions, resulting in a layer parallel to (001) with the adamantane groups located on its surface so that adjacent layers form only C—H⋯H—C contacts. The carbonate anions occupy special positions of 32 symmetry, whereas the adamantan-1-aminium cations occupy special positions of 3 symmetry.

## Related literature

For related structures, see: de Vries *et al.* (2011 ▶); Mullica *et al.* (1999 ▶); He & Wen (2006 ▶); Liu *et al.* (2009 ▶); Zhao *et al.* (2003 ▶). For applications of adamantane–ammonium salts in virology, see: Hoffmann (1973 ▶); Dolin *et al.* (1982 ▶); Bright *et al.* (2005 ▶); Betakova (2007 ▶). For applications of amines for the capture of CO_2_ from the atmosphere, see: Yang *et al.* (2008 ▶).
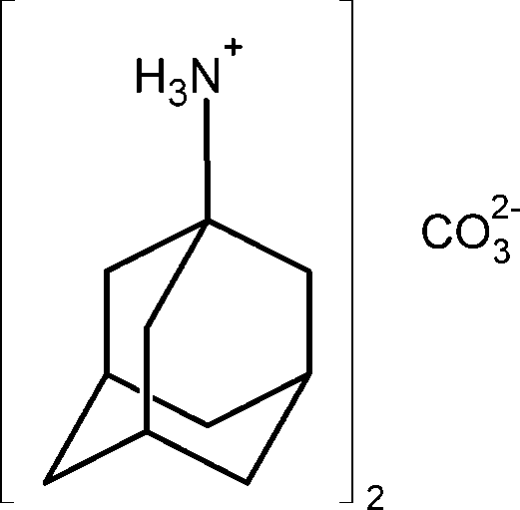


## Experimental

### 

#### Crystal data

2C_10_H_18_N^+^·CO_3_^2−^
*M* *_r_* = 364.52
Trigonal, 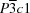

*a* = 6.4340 (6) Å
*c* = 25.474 (2) Å
*V* = 913.25 (14) Å^3^
*Z* = 2
Mo *K*α radiation
μ = 0.09 mm^−1^
*T* = 173 K
0.30 × 0.22 × 0.08 mm

#### Data collection

Bruker APEXII CCD diffractometer
3187 measured reflections
629 independent reflections
493 reflections with *I* > 2σ(*I*)
*R*_int_ = 0.062

#### Refinement

*R*[*F* ^2^ > 2σ(*F* ^2^)] = 0.042
*wR*(*F* ^2^) = 0.107
*S* = 1.08
629 reflections
45 parameters
H atoms treated by a mixture of independent and constrained refinement
Δρ_max_ = 0.22 e Å^−3^
Δρ_min_ = −0.18 e Å^−3^

### 

Data collection: *APEX2* (Bruker, 2005 ▶); cell refinement: *SAINT-Plus* (Bruker, 2005 ▶); data reduction: *SAINT-Plus* and *XPREP* (Bruker 2005 ▶); program(s) used to solve structure: *SHELXS97* (Sheldrick, 2008 ▶); program(s) used to refine structure: *SHELXL97* (Sheldrick, 2008 ▶); molecular graphics: *ORTEP-3 for Windows* (Farrugia, 1997 ▶), *Mercury* (Macrae *et al.*, 2008 ▶) and *PLATON* (Spek, 2009 ▶); software used to prepare material for publication: *WinGX* (Farrugia, 1999 ▶) and *PLATON*.

## Supplementary Material

Crystal structure: contains datablock(s) global, I. DOI: 10.1107/S1600536812011828/gk2467sup1.cif

Structure factors: contains datablock(s) I. DOI: 10.1107/S1600536812011828/gk2467Isup2.hkl

Supplementary material file. DOI: 10.1107/S1600536812011828/gk2467Isup3.cml

Additional supplementary materials:  crystallographic information; 3D view; checkCIF report

## Figures and Tables

**Table 1:** Hydrogen-bond geometry (Å, °)

*D*—H⋯*A*	*D*—H	H⋯*A*	*D*⋯*A*	*D*—H⋯*A*
N1—H1⋯O1^i^	0.999 (16)	1.778 (15)	2.764 (1)	168.7 (18)

Symmetry code: (i) 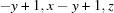.

**Fig. 1.**
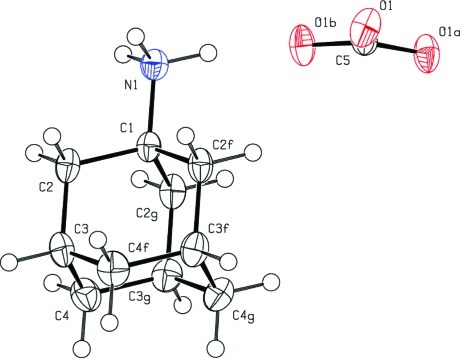
The molecular structure of the title compound. Displacement ellipsoids are shown at the 50% probability level. The atoms C2f to C4f are generated by the symmetry (1-y,x-y,z); C2g to C4g by (1-x+y, 1-x,z); O1a by (-y,x-y,z) and O2b by (-x+y,-x,z).

**Fig. 2.**
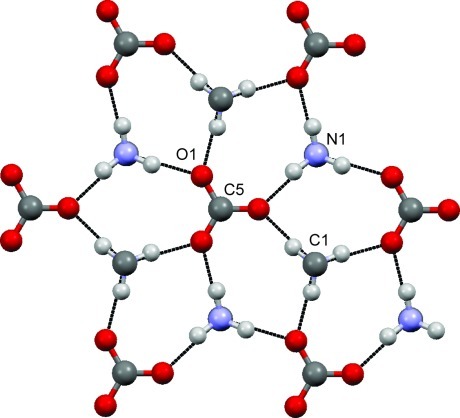
Intermolecular N—H···O hydrogen bonded (dashed lines) layers along [001] showing only the C-NH3 and CO3 groups for clarity.

**Fig. 3.**
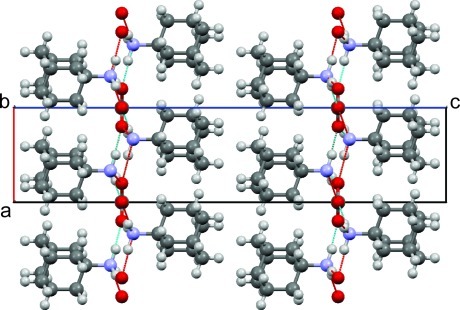
A view down the b axis of the unit cell of the title compound showing the hydrogen bonded layers. Notice that the carbonate ions occupy sites with 32 symmetry whereas cations occupy the sites of 3 symmetry.

## supplementary crystallographic information

### Comment

It has been reported that 1-aminoadamantane hydrochloride (marketed as Symmetrel) is effective in the prevention and treatment of the influenza (A) virus (Hoffmann, 1973; Dolin *et al.*, 1982; Bright *et al.*, 2005). However recent studies suggest that the virus is becoming increasingly resistant to this anti-influenza drug (Betakova, 2007).

In an attempt to crystallize pure 1-aminoadamantane from ethanol we obtained instead adamantan-1-aminium carbonate, illustrated in Fig. 1, suggesting that the amine had captured atmospheric CO_2_. We report the structure here. It is known that organic amines can trap CO_2_ as the ammonium carbonate salt and this property is being explored as a way to capture carbon dioxide from the atmosphere (Yang *et al.*, 2008).

Each carbonate ion of the title compound forms hydrogen bonds to six adamantane-ammonium ions, as shown in Fig. 2, forming a two-dimensional layer of adamantan-1-aminium carbonates parallel to (001). The hydrophobic adamantane layers interact with the neighbouring layers of adamantane-ammonium molecules *via* C—H···H–C contacts (see Fig. 3).

It is noted here that the structure of adamantan-1-aminium bicarbonate (Liu *et al.*, 2009) reported in the literature is isomorphous to adamantan-1-aminium nitrate (Zhao *et al.*, 2003). The former structure has unusually short H···H intermolecular contacts between NH_3_^+^ group H atom and bicarbonate H atom of 1.50 Å In addition the geometry of the hydrogen carbonate ion is very similar to that of the nitrate ion. A re-investigation of these structures is warranted.

### Experimental

Crystals were grown by slow evaporation of an ethanol solution of the title compound, 0.500 g in 10 ml of ethanol, and afforded colourless plates after three days under ambient conditions. Crystals decompose, with an emission of gas bubbles (presumably CO_2_), at 423–428 K.

### Refinement

The N-bound H atom was placed according to the observed electron density and was allowed to refine freely. The remaining H atoms were positioned geometrically and allowed to ride on their respective parent atoms, with C—H bond lengths of 1.00 (methine) and 0.99 Å (methylene CH_2_) and with *U*_iso_(H) = 1.2 times *U*_eq_(C).

### Crystal data

**Table:**

2C_10_H_18_N^+^·CO_3_^2^^−^	*D*_x_ = 1.326 Mg m^−^^3^
*M**_r_* = 364.52	Mo *K*α radiation, λ = 0.71069 Å
Trigonal, *P*3*c*1	Cell parameters from 819 reflections
Hall symbol: -P 3 2"c	θ = 3.2–25.8°
*a* = 6.4340 (6) Å	µ = 0.09 mm^−^^1^
*c* = 25.474 (2) Å	*T* = 173 K
*V* = 913.25 (14) Å^3^	Prism, colourless
*Z* = 2	0.30 × 0.22 × 0.08 mm
*F*(000) = 400	

### Data collection

**Table:**

Bruker APEXII CCD diffractometer	*R*_int_ = 0.062
Graphite monochromator	θ_max_ = 26.4°, θ_min_ = 1.6°
φ and ω scans	*h* = −8→5
3187 measured reflections	*k* = −2→8
629 independent reflections	*l* = −31→31
493 reflections with *I* > 2σ(*I*)	

### Refinement

**Table:**

Refinement on *F*^2^	Primary atom site location: structure-invariant direct methods
Least-squares matrix: full	Secondary atom site location: difference Fourier map
*R*[*F*^2^ > 2σ(*F*^2^)] = 0.042	Hydrogen site location: inferred from neighbouring sites
*wR*(*F*^2^) = 0.107	H atoms treated by a mixture of independent and constrained refinement
*S* = 1.08	*w* = 1/[σ^2^(*F*_o_^2^) + (0.0587*P*)^2^ + 0.010*P*] where *P* = (*F*_o_^2^ + 2*F*_c_^2^)/3
629 reflections	(Δ/σ)_max_ < 0.001
45 parameters	Δρ_max_ = 0.22 e Å^−^^3^
0 restraints	Δρ_min_ = −0.18 e Å^−^^3^

### Special details

**Table:**

Geometry. All e.s.d.'s (except the e.s.d. in the dihedral angle between two l.s. planes) are estimated using the full covariance matrix. The cell e.s.d.'s are taken into account individually in the estimation of e.s.d.'s in distances, angles and torsion angles; correlations between e.s.d.'s in cell parameters are only used when they are defined by crystal symmetry. An approximate (isotropic) treatment of cell e.s.d.'s is used for estimating e.s.d.'s involving l.s. planes.
Refinement. Refinement of *F*^2^ against ALL reflections. The weighted *R*-factor *wR* and goodness of fit *S* are based on *F*^2^, conventional *R*-factors *R* are based on *F*, with *F* set to zero for negative *F*^2^. The threshold expression of *F*^2^ > σ(*F*^2^) is used only for calculating *R*-factors(gt) *etc*. and is not relevant to the choice of reflections for refinement. *R*-factors based on *F*^2^ are statistically about twice as large as those based on *F*, and *R*- factors based on ALL data will be even larger.

### Fractional atomic coordinates and isotropic or equivalent isotropic displacement parameters (Å^2^)

**Table:**

	*x*	*y*	*z*	*U*_iso_*/*U*_eq_	
C1	0.6667	0.3333	0.16486 (9)	0.0236 (6)	
C2	0.9213 (2)	0.5034 (2)	0.14518 (6)	0.0278 (4)	
H2A	1.032	0.4505	0.1584	0.033*	
H2B	0.977	0.6678	0.1583	0.033*	
C3	0.9215 (2)	0.5033 (3)	0.08498 (6)	0.0309 (4)	
H3	1.0877	0.614	0.0719	0.037*	
C4	0.8365 (3)	0.2482 (3)	0.06502 (6)	0.0346 (4)	
H4A	0.838	0.2474	0.0262	0.042*	
H4B	0.9464	0.1932	0.0777	0.042*	
C5	0	0	0.25	0.0219 (7)	
N1	0.6667	0.3333	0.22372 (8)	0.0278 (5)	
O1	0	0.1993 (2)	0.25	0.0343 (4)	
H1	0.732 (4)	0.502 (3)	0.2358 (7)	0.060 (5)*	

### Atomic displacement parameters (Å^2^)

**Table:**

	*U*^11^	*U*^22^	*U*^33^	*U*^12^	*U*^13^	*U*^23^
C1	0.0198 (8)	0.0198 (8)	0.0312 (12)	0.0099 (4)	0	0
C2	0.0195 (8)	0.0205 (7)	0.0415 (9)	0.0087 (6)	−0.0018 (6)	−0.0014 (6)
C3	0.0217 (8)	0.0262 (8)	0.0404 (9)	0.0085 (6)	0.0061 (6)	0.0039 (6)
C4	0.0317 (9)	0.0350 (9)	0.0397 (8)	0.0185 (8)	0.0055 (6)	−0.0016 (7)
C5	0.0214 (10)	0.0214 (10)	0.0228 (15)	0.0107 (5)	0	0
N1	0.0253 (7)	0.0253 (7)	0.0328 (11)	0.0127 (3)	0	0
O1	0.0299 (8)	0.0221 (6)	0.0536 (10)	0.0149 (4)	−0.0091 (7)	−0.0045 (3)

### Geometric parameters (Å, º)

**Table:**

C1—N1	1.500 (3)	C3—C4	1.534 (2)
C1—C2	1.5295 (14)	C3—H3	1
C2—C3	1.5335 (19)	C4—H4A	0.99
C2—H2A	0.99	C4—H4B	0.99
C2—H2B	0.99	C5—O1	1.2820 (13)
C3—C4^i^	1.532 (2)	N1—H1	0.999 (16)
			
N1—C1—C2	109.13 (9)	C2—C3—C4	109.40 (12)
C2^ii^—C1—C2	109.81 (9)	C2—C3—H3	109.5
C1—C2—C3	109.18 (12)	C4—C3—H3	109.5
C1—C2—H2A	109.8	C3^ii^—C4—C3	109.57 (13)
C3—C2—H2A	109.8	C3—C4—H4A	109.8
C1—C2—H2B	109.8	C3—C4—H4B	109.8
C3—C2—H2B	109.8	H4A—C4—H4B	108.2
H2A—C2—H2B	108.3	O1^iii^—C5—O1	120
C4^i^—C3—C2	109.29 (11)	C1—N1—H1	107.9 (11)
C4^i^—C3—C4	109.58 (14)		
			
N1—C1—C2—C3	179.92 (8)	C1—C2—C3—C4	−59.84 (12)
C2^ii^—C1—C2—C3	60.34 (13)	C4^i^—C3—C4—C3^ii^	−59.76 (18)
C2^i^—C1—C2—C3	−60.50 (13)	C2—C3—C4—C3^ii^	60.04 (15)
C1—C2—C3—C4^i^	60.13 (13)		

Symmetry codes: (i) −*y*+1, *x*−*y*, *z*; (ii) −*x*+*y*+1, −*x*+1, *z*; (iii) −*y*, *x*−*y*, *z*.

### Hydrogen-bond geometry (Å, º)

**Table:**

*D*—H···*A*	*D*—H	H···*A*	*D*···*A*	*D*—H···*A*
N1—H1···O1^iv^	0.999 (16)	1.778 (15)	2.7644 (11)	168.7 (18)

Symmetry code: (iv) −*y*+1, *x*−*y*+1, *z*.
